# Optimization of Cultivation Parameters for Scale-Up Production of *Streptomyces recifensis* SN1E1

**DOI:** 10.4014/jmb.2602.02031

**Published:** 2026-04-21

**Authors:** Su In Lee, Youn-Sig Kwak

**Affiliations:** 1 Division of Applied Life Science, Gyeongsang National University, Jinju 52828, Republic of Korea; 2 Research Institute of Life Science, Gyeongsang National University, Jinju 52828, Republic of Korea

**Keywords:** Biological control, Formulation, Plant growth-promoting rhizobacteria, *Streptomyces*

## Abstract

This study evaluated the growth characteristics and formulation stability of *Streptomyces recifensis* SN1E1, a potent antagonist against the apple fire blight pathogen *Erwinia amylovora*, and to establish optimal conditions for its large-scale cultivation and shelf-life formulation for sustainable agricultural use. We investigated specific carbon sources and formulation matrices to enhance growth, sporulation, and storage viability. Experimental results indicated that the addition of D-mannitol significantly expedited spore formation and promoted the overall growth of strain SN1E1. Furthermore, to ensure product stability, various preservative concentrations and formulation bases were assessed. Among the formulations tested, a mixture containing 0.02% soya and 5% skim milk exhibited the highest bacterial cell survival rate under storage conditions. The study suggested that D-mannitol is a favorable carbon source for the efficient sporulation of *S. recifensis* SN1E1 and confirms that a soya/skim milk-based formulation significantly improves the stability of the final product. These protocols provide a reliable foundation for the commercialization of this biocontrol agent. This study offers essential technical insights into the mass cultivation and preservation of plant growth-promoting *Streptomyces* species, facilitating their practical application in combating crop diseases like apple fire blight.

## Introduction

With the world's population projected to reach approximately 9.7 billion by 2050 and an anticipated decline in climate-related crop productivity, stable and sustainable agriculture is increasingly recognized as a critical issue for food security [1-3]. According to the Organization for Economic Cooperation and Development, achieving a stable and sustainable food supply now requires a productivity increase of over 1.5-fold compared to current levels [4]. These perspectives have reinforced the need for a green revolution in agriculture, introducing the concept of the phytobiome [5]. The phytobiome encompasses all factors related to crop cultivation, where microbial and other environmental factors work synergistically in a mutually beneficial manner to protect plants and enhance productivity [6]. Plant-associated microbes utilize every tissue of the plant as a habitat for survival, from the soil to the plant's aerial parts, and are classified into the rhizosphere, endosphere, phyllosphere, and seedbiome [7-10]. Additionally, plant growth-promoting rhizobacteria (PGPR) have been extensively studied over the past decades and are known to partially alleviate damage caused by biotic and abiotic stresses [11]. PGPR employ various pathways, such as indole-3-acetic acid production, siderophore synthesis, nitrogen fixation, and the production of antibiotic metabolites, to enhance plant growth and protect against various diseases. Representative microbial groups of PGPR include *Pseudomonas*, *Bacillus*, and *Streptomyces*, which are known for their direct and indirect mechanisms that enhance plant growth [12-15].

The genus *Streptomyces* is reported to form clusters in the rhizosphere, endosphere, and phyllosphere of plants [16, 17]. Additionally, it is recognized as a key source of biologically active antibiotics and beneficial secondary metabolites [18]. To date, 70–80% of known antibiotics have been isolated and reported from the genus *Streptomyces* [19, 20]. According to previous research, the transformation of secondary metabolites occurs during the stage of spore formation from aerial hyphae in the life cycle of *Streptomyces* [21]. Therefore, spore formation is considered a crucial process in the production of antibiotics by *Streptomyces*. *S. recifensis* SN1E1 demonstrated remarkable antagonistic properties by inhibiting the growth of *Erwinia amylovora*, the pathogen causing apple fire blight disease. Through genome sequencing of SN1E1, a CRISPR/Cas9 system was utilized to create mutants targeting biosynthetic genes associated with antibacterial activity. The loss-of-function mutants showed a reduction in antibacterial activity against the pathogen responsible for fire blight disease. Furthermore, extensive plant growth-promoting tests revealed low protease activity, indicating harmlessness to bees. The strain exhibited antibacterial activity against bee gut pathogens and demonstrated high attachment and sporulation capabilities [9].

Functional beneficial microorganisms that effectively inhibit the occurrence of plant diseases require mass cultivation processes for practical application [22, 23]. However, pure cultivation of microorganisms often involves the use of expensive experimental media, posing limitations for field applications in agriculture [24]. Additionally, the commercialization of biocontrol agents requires various processes to showcase their activity. Moreover, *Streptomyces* species are known to be quite challenging to mass-culture, emphasizing the need to establish optimal growth conditions [25]. In the mass cultivation of agricultural microorganisms, the careful selection of carbon and nitrogen sources is crucial for promoting the growth of the target microorganism. Factors such as the acidity of the medium, cultivation temperature, and rotational speed of the cultivation apparatus also need consideration [23]. This study aimed to elucidate substances specifically involved in microbial growth and spore formation using the SN1E1 strain. Additionally, we sought to select preservatives that enhance the stability of the formulation. Based on fundamental research identifying the carbon and nitrogen sources and preservatives essential for the mass production process of the selected beneficial microorganism, the presented results establish foundational data for the development of biological control *Streptomyces* mass production.

## Materials and Methods

### Media Conditions

To observe growth differences in various media, the strain cultivation was performed on ISP-1 broth (Casein enzymic hydrolysate 5 g, Yeast extract 3 g per L, pH 7.0), ISP-2 broth (Peptone 5 g, Yeast extract 3 g, Malt extract 3 g, Dextrose 10 g per L, pH 6.2), PDK broth (Potato dextrose broth 10 g, Peptone 10 g per L), R2A broth (Yeast extract 0.5 g, Proteose peptone 0.5 g, Casamino acids 0.5 g, Glucose 0.5 g, Soluble starch 0.5 g, Na-pyruvate 0.3 g, K_2_HPO_4_ 0.3 g, MgSO_4_·7H_2_O 0.05 g per L), and TSB medium (Tryptic soy broth 30 g per L). Initially, the strain was cultured on MS (D-mannitol 20 g, Soya flour 20 g, Agar 20 g per L) medium at 28°C for 5 days. After 5 days, the produced spores were inoculated into test tubes containing 5 mL of each medium and cultured at 28°C for an additional 3 days. Subsequently, the culture was transferred to 200 mL flasks and incubated in a shaking incubator (200 rpm) at 28°C for 7 days. Afterward, 1 mL of the culture was mixed with 9 mL of sterile water, and this suspension was further diluted to 10^-6^. Bacterial cell density was then measured using the dilution plate method on PDK medium. Additionally, the presence of spores and the degree of clumping were compared under a microscope (CX23, Olympus, Japan).

### Screening carbon and nitrogen sources

*S. recifensis* SN1E1 was cultured on MS medium at 28°C for 5 days. A single colony was streaked onto a fresh MS plate, and mature spores were collected after 7 days using 1 mL of ddH_2_O with a sterilized cotton ball. Following filtration, the spore concentration was adjusted to an OD_600_ nm of 2.0, mixed with a stock solution, and incubated in sealed plates: PM1 for carbon sources and PM3B for nitrogen sources (Biolog, Germany) at 28°C for 2 days. Subsequently, 10 μL of Biolog redox dye (100 x) was added to each well, and the color change intensity was monitored at OD_570_ nm every minute for 72 h using a Synergy H1 Hybrid Multi-Mode microplate reader (BioTek, USA). The selection was based on the highest values obtained from Spearman correlation analysis. To assess the impact of the selected carbon and nitrogen sources on the growth of the SN1E1 strain, the strain was cultured in a medium containing the selected sources. Cultivation was carried out in flasks with baffles in a shaking incubator at 200 rpm and 28°C for 7 days. Post-cultivation, mycelium and spores were observed through microscopic analysis. Additionally, 1 mL of the culture was mixed with 9 mL of sterile water, and this suspension was further diluted to 10^-6^. Subsequently, bacterial cell density was measured using the dilution plate method on PDK medium.

### Optimization of pH and Temperature Condition

To determine the optimal pH for the *S. recifensis* SN1E1 strain, various levels (pH 2, 5, 7, 7.2, 10) were adjusted, and the growth of the strain was measured at OD_600_ nm using a Synergy H1 Hybrid Multi-Mode microplate reader at 28°C for 72 h. The growth graphs at different pH conditions were visualized using the R program (ver 4.1.3). To determine the optimal growth temperature for the strain, temperature ranges of 25–30°C were divided into 25, 28, and 30°C. The growth medium used was the same as in the optimal pH adjustment experiment. The experiments were conducted using a Bioreactor system (RTC-1C, λ = 850 nm – bioreactor fixed value) (Biosan, Latvia). The working volume was set at 25 mL, with conditions of 1,000 rpm, a 60-sec revolution spin period, and a time period of 72 h. After cultivation, microscopic analysis was conducted to observe mycelium and spores. Additionally, 1 mL of the culture was mixed with 9 mL of sterile water, and this suspension was further diluted to 10^-6^. Subsequently, bacterial cell density was measured using the dilution plate method on PDK medium.

### Optimization of Freeze-Drying Conditions

To establish long-term storage conditions for *S. recifensis* SN1E1, we first determined the efficiency of anti-freezing agents. During freeze-drying, polymers such as skim milk and starch, as well as low-molecular-weight substances like glucose and lactose, were amended as anti-freezing agents. To assess the preservative effect of different skim milk concentrations, concentrations ranging from 3% to 10% were prepared. These were then mixed 1:1 with cultured cells to achieve final concentrations of 1.5%, 2.5%, and 5%. For the cultured cells, the culture medium from flasks containing the strain was subjected to centrifugation at 8,000 rpm for 20 min. After mixing, the *S. recifensis* SN1E1 suspension was transferred into a 50 mL tube (SPL, Republic of Korea) and pre-frozen at -80°C for 24 hours. Subsequently, the tube was stored at -55°C for 72 h in a vacuum freeze dryer HyperCool (Gyrozen Co. Ltd., Republic of Korea). Following drying, 1 g of the dried product was mixed with 9 mL of sterile water, and this suspension was further diluted to 10^-6^. The density at different concentrations of skim milk was measured using the dilution plate method on PDK medium.

Secondly, we determined the concentration of soya required to enhance spore production during the cultivation of *S. recifensis* SN1E1. Soya is a key component in selective media for *Streptomyces* and is known to support bacterial growth. To compare spore production, different concentrations of soya ranging from 0.02% to 1% were prepared. Subsequently, spore production was compared by mixing the cultures with concentrations of 0.02%, 0.2%, and 1% in the agar medium, along with a control group without soya. After cultivating for 10 days at 28°C in a shaking incubator (200 rpm), the samples were subjected to freeze-drying at -55°C for 72 h. Post-drying, 1 g of the dried product was mixed with 9 mL of sterile water, and this suspension was further diluted to 10^-6^. Bacterial density for various concentrations of soya was measured on PDK agar using the dilution plate method. Spore production was quantified through microscopic analysis at 1,000-fold magnification for comparative assessment.

For the third step, we determined the concentrations of dextrose and soluble starch as enhancers to improve the preservation rate of SN1E1 after freeze-drying and storage at a high temperature. Dextrose and starch act as cryoprotectants, reducing temperature and moisture loss during freeze-drying. However, since these chemicals can also function as enhancers, varying concentrations in the range of 5–10 g were prepared to observe preservation rates under different conditions. Next, we divided the freeze-dried samples, using 3 g as the base, into combinations of 5 g dextrose with 5 g soluble starch, and 10 g dextrose with 10 g soluble starch. After mixing, the samples were stored in a 52°C incubator for 4 weeks. Following the storage period, 1 g of the product was mixed with 9 mL of sterile water weekly, diluting this suspension to 10^-6^. The preservation rates for different concentrations were measured using the dilution plate method on PDK agar plates.

### Monitoring the Survival of *S. recifensis* SN1E1

To assess the viability of *S. recifensis* SN1E1 in each freeze-drying experiment, samples were collected before (cell-down samples and a 1:1 suspension with sterile water) and after (powdered state post-drying) the freeze-drying process. The cell concentration in the pre-freeze-dried suspension or post-dried powder samples was measured using the plate count method. The dried *S. recifensis* SN1E1 powder was transferred to a 50 mL conical tube (SPL) for survival rate assessment at a high temperature (52°C). The transferred powder was measured for surviving cell counts at 2-week intervals for 4 weeks in a 52°C shaking incubator. After appropriate dilution, 100 μL was spread on PDK agar plates and incubated at 28°C for 3–5 days. Colony counts on plates containing 1 to 1,000 colonies were used to calculate the viable cell count in each sample.

## Results

### Growth Media and Sporulation Condition of *S. recifensis* SN1E1

**Fig. 1:**
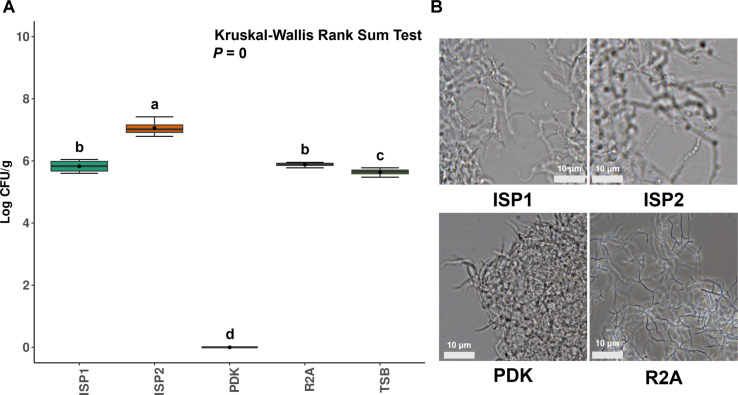
Influence of various culture medium by *S. recifensis* SN1E1. (**A**) A graph depicting CFU measurements based on various media, including ISP1 broth, ISP2 broth, PDK broth, R2A broth, and TSB. (**B**) Aerial hyphae and spore chain structure were visualized under a 1,000 x microscope (scale bars = 10 μm).

To determine the optimal culture conditions for enhancing the initial growth rate during mass cultivation, we tested ISP1 and ISP2 broth media, which are commonly used for SN1E1, as well as the previously used PDK agar medium. Additionally, we included R2A and TSB broth media, commonly used for bacterial cultivation. The growth rate of the SN1E1 strain was assessed by measuring colony-forming units (CFU) and sporulation ability (Fig. 1). The results showed that, excluding PDK, the SN1E1 strain demonstrated CFU values ranging from 10^6^ to 10^7^ on the other four media (Fig. 1A). The highest growth rate was observed on ISP2 broth medium, and consistent with the sporulation test, SN1E1 exhibited optimal growth when yeast extract and mannitol were used as the main carbon sources. However, on PDK broth medium, lower CFU values were observed due to aggregation phenomena, unlike in the other media. Furthermore, when assessing sporulation ability, high-growth-rate media such as ISP1, ISP2, and PDK broth showed limited sporulation (Fig. 1B). Interestingly, R2A broth medium exhibited sporulation with a count of 10^5^ CFU/mL. For ISP1, ISP2, and PDK broth media, the aggregation phenomenon suggested inadequate sporulation under these conditions.

### pH and Temperature Optimization

The growth of microorganism is significantly influenced by pH conditions, and if growth of a certain stain is possible at a specific pH, unintentional contamination can be effectively avoided. To determine the optimal pH conditions that could enhance the initial growth rate during mass cultivation, pH values of 2, 5, 7, 7.2, and 10 were tested. The growth rate and specific growth rate (calculated using the slope for maximum growth rate) of SN1E1 were measured. The results revealed that the SN1E1 strain exhibited growth under neutral conditions (pH 7, pH 7.2), acidic conditions (pH 5), and alkaline conditions (pH 10). The fastest growth rate was observed under neutral conditions (pH 7.2 and pH 7.0). Additionally, SN1E1 demonstrated growth at pH 7, pH 7.2, and pH 10, with the highest growth rate observed at the neutral pH of 7.2 (Fig. S1).

To determine the optimal temperature conditions for enhancing growth rates during large-scale cultivation, we tested temperatures of 25, 28, and 30°C. Setting the optimal temperature for microbial culture is essential for minimizing the culture period and improving the efficiency of the culture medium. Cell density (measured as absorbance at 850 nm) was monitored every hour for 72 hours (Fig. S2). The results revealed rapid growth at 10 hours for all three temperatures. Subsequently, growth reached its peak at 38 hours for 25°C, 35 h for 28°C, and 33 h for 30°C. While all three temperatures exhibited significant growth after 10 hours, the fastest growth rate was observed at 30°C. Furthermore, at the point of peak growth, the optical densities were OD_850_ 2.87 for 25°C, OD_850_ 2.9 for 28°C, and OD_850_ 3.76 for 30°C (Fig. S2). Notably, the 30°C condition displayed a higher OD value compared to the 25 and 28°C conditions, indicating superior growth under the 30°C temperature regime.

### Carbon and Nitrogen Source Preference Verification

**Fig. 2:**
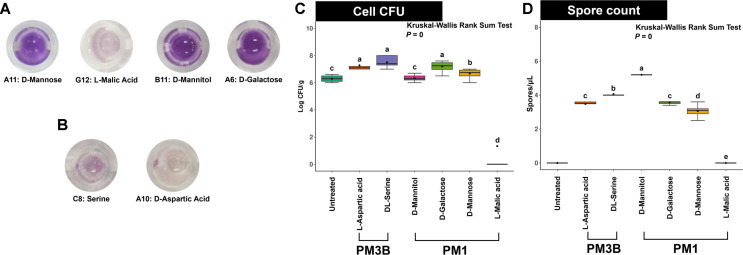
Biolog phenotype array for carbon and nitrogen utilization and bacterial growth. Biolog plates (*n* = 3, 3 independent experiments) were inoculated with strain SN1E1 (OD_600nm_ 0.2) and incubated at 28°C for 72 h. Redox dye mix MA was added when the second or the fourth wells in a row turned yellow and a color change to purple was observed at 37°C. The color was assayed at 570 nm. (**A**) PM1 plate for carbon sources. (**B**) PM3B plate for nitrogen sources. (**C**) Measurement of SN1E1 CFU in R2A broth medium. (**D**) Spore counts of strain SN1E1. Box plot represent Kruskal-Wallis Rank Sum Test, *P* value less than 0.05.

To select carbon and nitrogen sources affecting the growth of the SN1E1 strain, Biolog PM1 plates (carbon sources) and PM 3B plates (nitrogen sources) were employed in the experiment. The results revealed that SN1E1 exhibited growth on single carbon sources such as D-Mannose, L-Malic acid, D-Mannitol, and D-Galactose. For nitrogen sources, growth was observed with D-Serine and D-Aspartic acid (Table 1). Each selection was based on a cutoff at the “very strong” to “strong” levels according to False Discovery Rate (FDR) values (Fig. 2A and 2B). When examining the growth rates based on the selected carbon and nitrogen sources, SN1E1 maintained a growth rate in the range of 10^6^–10^8^ Log CFU/g for all sources except Malic acid (Fig. 2C). Using the four selected carbon sources and two nitrogen sources, we conducted validation experiments in liquid culture conditions to assess their involvement in *Streptomyces* sporulation and secondary metabolite formation. The results indicated that SN1E1 did not exhibit robust sporulation with any of the nitrogen sources, while vigorous sporulation was observed in the presence of D-Mannitol among the carbon sources. Interestingly, the addition of L-Malic acid resulted in the absence of mycelial formation, indicating a lack of sporulation. Consequently, this observation implies a low CFU in the culture (Fig. 2D).

### Optimal Freeze-Drying Conditions

**Fig. 3:**
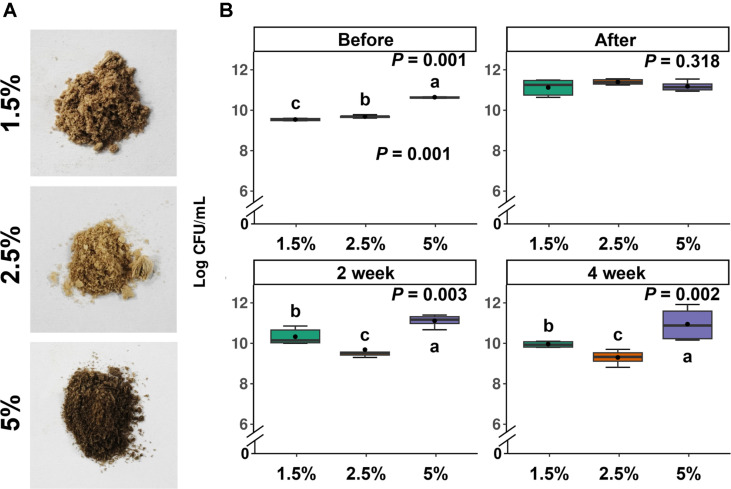
Optimization of freeze-drying preservative. (**A**) The morphological appearance after freeze-drying at different concentrations of skim milk. (**B**) CFU after a survival test at high temperature (52°C). SN1E1 cells were mixed with skim milk at varying concentrations and subjected to freeze-drying at -55°C for 72 h. The density at different concentrations of skim milk was measured using the dilution plate method on PDK agar medium. Box plot represent Kruskal-Wallis Rank Sum Test, *p* < 0.05.

To establish the conditions for freeze-drying after mass cultivation, various conditions were tested. Initially, to determine the cryoprotectant conditions, skim milk concentrations in the range of 3%–10% were tested. Cells from the mass cultivation were mixed in a 1:1 ratio, resulting in final concentrations of 1.5%, 2.5%, and 5% (Fig. 3). As a result, before freeze-drying, 10^9^ CFU were observed at skim milk concentrations of 1.5% and 2.5%, while 5% showed 10^11^ CFU. After freeze-drying, all three concentrations showed 10^11^ CFU. Subsequently, survival rates were examined during a 4-week storage period at a high temperature (52°C). In the second week, the 5% concentration-maintained 10^11^ CFU, consistent with the post-freeze-drying count. However, at 1.5%, the count decreased to 10^10^ CFU, and at 2.5%, it dropped to 10^9^ CFU. In the fourth week, the 5% concentration-maintained 10^11^ CFU, similar to the second week, while 1.5% decreased to 10^9^ CFU, and 2.5% dropped to 10^8^ CFU. This led to the establishment of the optimal cryoprotectant concentration at a final concentration of 5% (Fig. 3B).

**Fig. 4:**
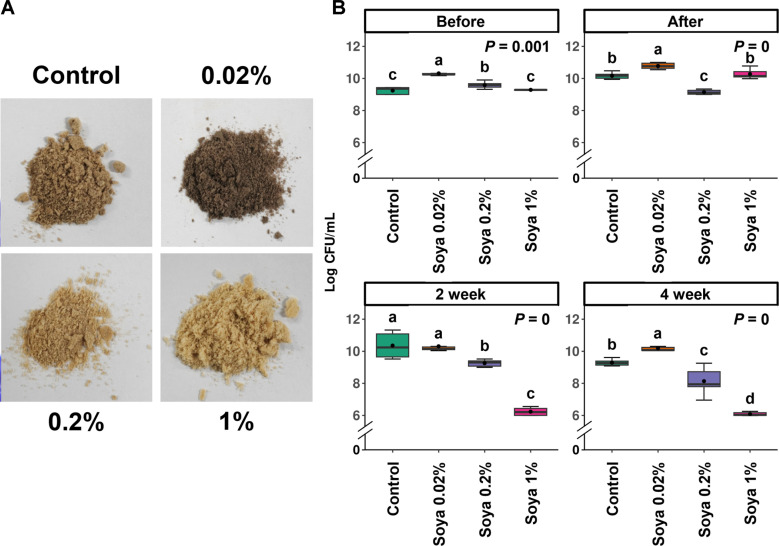
Optimization of soya concentration. (**A**) The morphological appearance after freeze-drying at different concentrations of soya. (**B**) CFU after a survival test at high temperature (52°C). SN1E1 cells were mixed with various concentrations of soya and subjected to freeze-drying at -55°C for 72 h. The density for different soya concentrations was measured using the dilution plate method on PDK agar medium. Box plots represented the Kruskal-Wallis Rank Sum Test, indicating significance with a *p*-value below 0.05.

Next, to enhance spore production during cultivation, we tested soya concentrations ranging from 0.02% to 1%. During mass culture, soya concentrations of 0%, 0.02%, 0.2%, and 1% were obtained in the medium based on volume (Fig. 4). As a result, before freeze-drying, 10^9^–10^10^ CFU were observed under all conditions, with the lowest count of 10^9^ CFU at 0.2% soya concentration immediately after freeze-drying. In contrast, the other concentrations showed an increase in CFU compared to before freezing, remaining at 10^10^ CFU. Similar to the previous experiment, the cultures were stored at a high temperature (52°C) for 4 weeks to assess survival rates. In the second week, the 1% soya concentration showed a decrease to 10^6^ CFU, while the other treatment groups-maintained 10^10^ CFU. In the fourth week, the 0.02% soya concentration-maintained 10^10^ CFU or higher, while the other concentrations showed a decrease. Additionally, when confirming spore formation, only the 0.02% soya concentration-maintained 10^8^ CFU, excluding the other concentrations. As a result, we established a soya concentration of 0.02% to consistently maintain spore viability and survival rates at high temperatures (Fig. 4B).

**Fig. 5:**
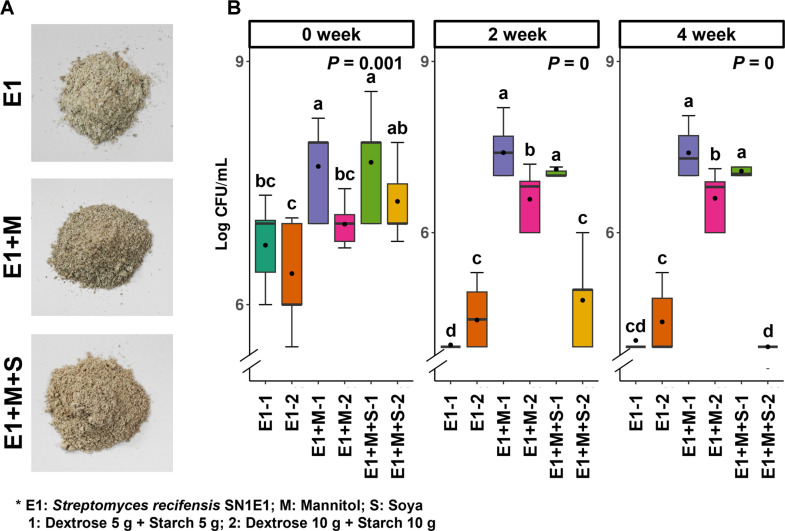
Optimization of dextrose and soluble starch concentrations. (**A**) The morphological appearance after freeze-drying at different concentrations of dextrose and soluble starch. (**B**) CFU after a survival test at high temperature (52°C). SN1E1 cells were freeze-dried at -55°C for 72 hours, mixed with various concentrations of dextrose and soluble starch, followed by a survival test at high temperature. CFU densities for different concentrations of dextrose and soluble starch were measured using the dilution plate method on PDK agar medium. Box plots represent the Kruskal-Wallis Rank Sum Test, indicating significance with a *p*-value below 0.05.

Finally, to enhance the preservation of SN1E1 after freeze-drying and storage at high temperatures, we tested the concentrations of dextrose and soluble starch. We divided the concentrations of dextrose and soluble starch into two categories and stored them at a high temperature (52°C) (Fig. 5). In the second week, when using 10 g of dextrose and 10 g of soluble starch, a decrease in CFU was observed in all treatment groups. Furthermore, the treatment groups without mannitol and soya showed a sharp decrease to 10^6^ CFU from the second week onwards. Entering the fourth week, a trend similar to the second week was observed, and the most effective stability at high temperatures was achieved when mannitol and soya were added to the SN1E1 strain before freeze-drying, followed by the addition of 5 g each of dextrose and soluble starch. As a result, the concentration of 5 g dextrose and 5 g soluble starch was established to maintain survival rates at high temperatures (Fig. 5B).

## Discussion

Fungicides are routinely used in agricultural systems to control various plant diseases [26]. However, the intensive use of fungicides has led to the emergence and development of fungicide-resistant pathogen populations, causing a decline in the effectiveness of disease control [27, 28]. Moreover, the consequences include decreased biodiversity, biological accumulation of chemicals, farmer poisoning, and the rapid emergence of pesticide-resistant pathogens. Therefore, the use of BCAs for plant disease management has become a rapidly evolving research area, considering its potential impact on plant productivity, animal health, and human health.

BCAs are deemed essential for agricultural systems globally because consumers worldwide recognize the need to significantly reduce the use of chemical substances in agricultural production [9]. The concept of BCAs has been actively implemented for over a century, demonstrating its influence not only on various plant diseases but also on specific plant and microbial communities. However, for practical application, isolated BCAs require large-scale cultivation processes. Yet, pure cultivation of microorganisms remains challenging, especially considering the cost and limitations associated with using high-priced media suitable for on-field application [24]. Additionally, the commercialization of BCAs involves complex processes, and certain microbial strains, such as *Streptomyces*, possess characteristics that make them challenging to cultivate and sporulate, contributing to their currently limited market presence. Therefore, in this study, we utilized *S. recifensis* SN1E1, which demonstrated significant inhibitory effects against fire blight pathogens posing a major threat to apple production and cultivation. The SN1E1 strain exhibited outstanding antimicrobial effects on apple fruits, roses, apple flowers, and apple seedlings, demonstrating robust antifungal activity even in its supernatant. The proven capabilities of the SN1E1 strain highlight its potential as a novel biological control agent. Furthermore, we identified the essential carbon and nitrogen sources, sporulation conditions, and preservatives necessary for optimizing the large-scale production process, aiming to enhance the viability of practical applications.

The composition of the medium can significantly impact the secondary metabolite biosynthesis of microorganisms. Previous studies have recognized, manipulated, and optimized medium components to increase antibiotic production. For instance, Wang *et al*. [29] applied the Response Surface Methodology approach to optimize the medium for *Xenorhabdus bovienii*, resulting in a 37.8% increase in antibiotic activity. Similarly, Gan *et al*. [30] reported a 3.42-fold increase in antibiotic production by *Bacillus siamensis* A72 through medium optimization. Building on these findings, the growth of *S. recifensis* SN1E1 was assessed on various media, revealing that the R2A medium was suitable due to its high spore formation and low aggregation. Furthermore, the spore formation necessary for antibiotic production is known to be related to medium components. Previous research indicated that the addition of KBr and CaCO_3_ during cultivation increased antibiotic production [31]. Testing selected carbon and nitrogen sources identified through a Biolog plate test on the R2A medium revealed that the highest spore formation occurred when cultivated with D-Mannitol. In general, sporulation is involved the long-term stability of Actinomycetes, and in many cases, efficient sporulation is important in the culture of Actinomycetes because Actinomycetes produce various secondary metabolites during the spore formation period. This suggests that the spore-forming ability of the SN1E1 strain is associated with the carbon source.

Next, after establishing conditions to enhance growth rates and sporulation, we utilized the common freeze-drying method to attain a formulated, pelletized form. In the freeze-drying process, polymers such as skim milk, dextran, and polyvinylpyrrolidone (PVP) are utilized to minimize temperature and moisture loss [32, 33]. Additionally, low-molecular-weight substances like glucose, sucrose, lactose, and sodium glutamate are incorporated. Park *et al*. [34] reported a 63% survival rate for *P. temperata* M1021 when mixed with 7% skim milk. Building upon these findings, we assessed the preservation rates of *S. recifensis* SN1E1 with varying concentrations of skim milk. The results indicated that the highest preservation rate was achieved when freeze-dried with a 1:1 mixture of 10% skim milk. This aligns with previous research and offers the potential for universal application across diverse strains.

Through this study, we were able to establish conditions for culturing *Streptomyces* genera with challenging sporulation requirements. Additionally, we reaffirmed that high concentrations of preservatives do not necessarily enhance preservation rates. Moreover, we provided fundamental data on the mass production and formulation processes of *S. recifensis* SN1E1. In conclusion, we presented a pioneering process for establishing formulation conditions using *Streptomyces* genera and proposed a novel biocontrol factor for managing fire blight.

## Supplemental Materials

Supplementary data for this paper are available on-line only at http://jmb.or.kr.



## Figures and Tables

**Table 1:** Substrates in Biolog PM1 and PM3B MicroPlates metabolized by *S. recifensis* SN1E1.

Plate PM1				Plate PM3B			
Well	Chemical	FDR	Range^*^	Well	Chemical	FDR	Range
A11	D-Mannose	1.42E-09	very strong	C8	D-Serine	0.0019	Strong
G12	L-Malic Acid	4.45E-05	very strong	A10	D-Aspartic acid	0.0175	Strong
B11	D-Mannitol	6.39E-05	very strong				
A6	D-Galactose	1.36E-04	very strong				

^*^Very strong: p-value < 0.001; Strong: 0.001 ≤ *p*-value<0.01; Moderate: 0.01 ≤ *p*-value < 0.05; Week: 0.05 ≤ *p*-value
