# Supplementary material for: Optimization of Cultivation Parameters for Scale-Up Production of Streptomyces recifensis SN1E1
Source: J Microbiol Biotechnol. 2026 Apr 21;36:e2602031. doi: 10.4014/jmb.2602.02031 (PMC13102624; doi:10.4014/jmb.2602.02031)
Supplement: Supplementary file 1 [file jmb-36-e2602031-supple.pdf]

J. Microbiol. Biotechnol. 2026. 36: e2602031

https://doi.org/10.4014/jmb.2602.02031

Supplementary Figures

Optimization of Cultivation Parameters for Large-Scale Production of *Streptomyces recifensis* SN1E1

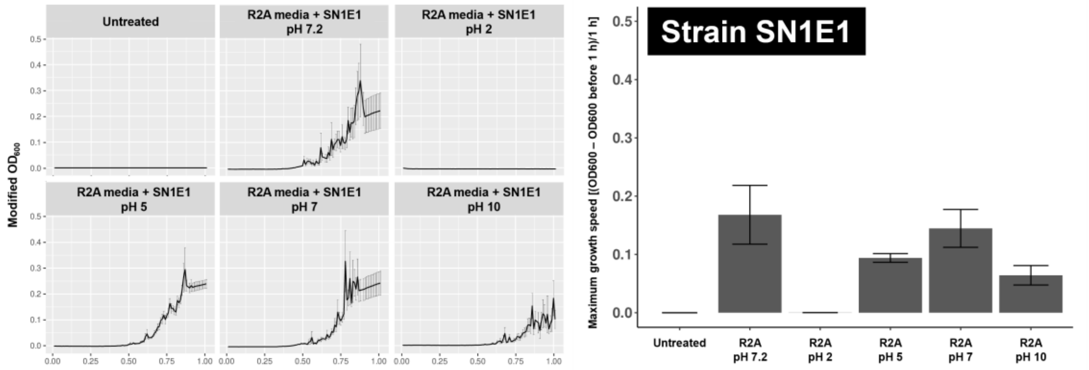

Fig. S1. Variation with pH in bacterial growth. The spore stock (10 μL) of the suspension was added to each well of a 96-well plate. The plates were incubated, and the optical density was measured with orbital shaking using a Synergy H1 hybrid Multi-Mode microplate reader for a period of 72 h at a temperature of 28°C. The left panel showed bacterial growth in each pH condition. The right panel presented the maximum growth speed of the SN1E1 strain.

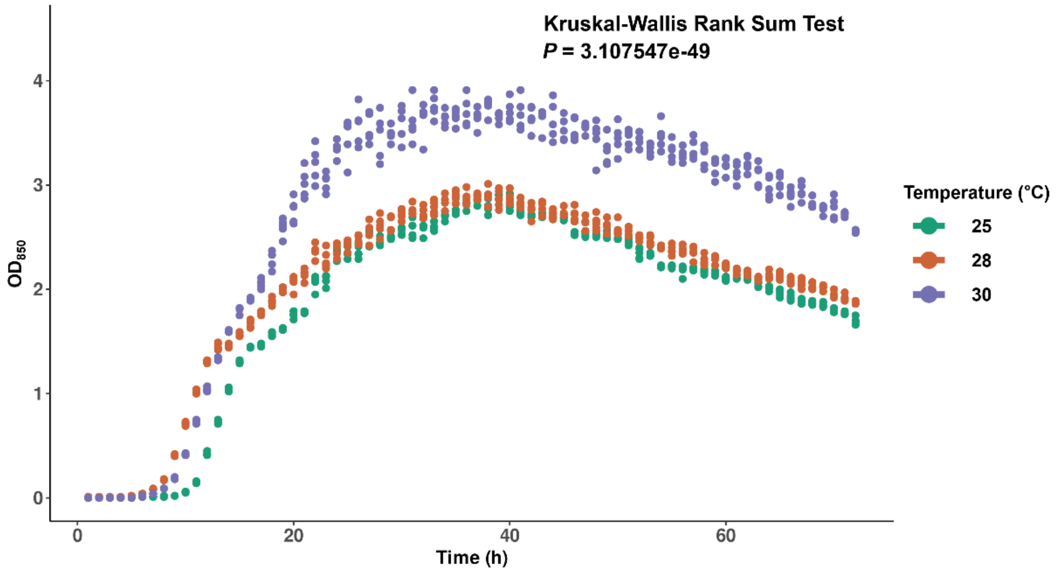

Fig S2. Variation with temperature in bacterial growth. Performed the experiment using a 25 mL mixed solution. The temperature was maintained at 25, 28, and 30°C, and the optical density was measured at 850 nm using RTC-1C over a period of 72 h. Line plot represent the Kruskal-Wallis Rank Sum Test, indicating significance with a p-value below 0.05
